# Soybean-derived Bowman-Birk inhibitor inhibits neurotoxicity of LPS-activated macrophages

**DOI:** 10.1186/1742-2094-8-15

**Published:** 2011-02-15

**Authors:** Jieliang Li, Li Ye, Denise R Cook, Xu Wang, Jinping Liu, Dennis L Kolson, Yuri Persidsky, Wen-Zhe Ho

**Affiliations:** 1 Department of Pathology & Laboratory Medicine, Temple University School of Medicine, Philadelphia, Pennsylvania, USA; 2 Department of Neurology, University of Pennsylvania School of Medicine, Philadelphia, Pennsylvania, USA; 3 Animal Biosafety Level 3 Laboratory, Wuhan University, Wuhan, 430071 PR China

## Abstract

**Background:**

Lipopolysaccharide (LPS), the major component of the outer membrane of gram-negative bacteria, can activate immune cells including macrophages. Activation of macrophages in the central nervous system (CNS) contributes to neuronal injury. Bowman-Birk inhibitor (BBI), a soybean-derived protease inhibitor, has anti-inflammatory properties. In this study, we examined whether BBI has the ability to inhibit LPS-mediated macrophage activation, reducing the release of pro-inflammatory cytokines and subsequent neurotoxicity in primary cortical neural cultures.

**Methods:**

Mixed cortical neural cultures from rat were used as target cells for testing neurotoxicity induced by LPS-treated macrophage supernatant. Neuronal survival was measured using a cell-based ELISA method for expression of the neuronal marker MAP-2. Intracellular reactive oxygen species (ROS) production in macrophages was measured via 2', 7'-dichlorofluorescin diacetate (DCFH_2_DA) oxidation. Cytokine expression was determined by quantitative real-time PCR.

**Results:**

LPS treatment of macrophages induced expression of proinflammatory cytokines (IL-1β, IL-6 and TNF-α) and of ROS. In contrast, BBI pretreatment (1-100 μg/ml) of macrophages significantly inhibited LPS-mediated induction of these cytokines and ROS. Further, supernatant from BBI-pretreated and LPS-activated macrophage cultures was found to be less cytotoxic to neurons than that from non-BBI-pretreated and LPS-activated macrophage cultures. BBI, when directly added to the neuronal cultures (1-100 μg/ml), had no protective effect on neurons with or without LPS-activated macrophage supernatant treatment. In addition, BBI (100 μg/ml) had no effect on N-methyl-D-aspartic acid (NMDA)-mediated neurotoxicity.

**Conclusions:**

These findings demonstrate that BBI, through its anti-inflammatory properties, protects neurons from neurotoxicity mediated by activated macrophages.

## Background

Inflammation plays a critical role in neurodegenerative diseases such as Parkinson's disease, multiple sclerosis, Alzheimer's disease, and HIV-associated dementia (HAD). Activation of microglia, the intrinsic macrophages in the central nervous system (CNS) [1], is a characteristic feature of most neurodegenerative diseases upon systemic infection. Mounting evidence indicates that macrophage/microglia activation contributes to inflammation and neuronal injury in a number of neurological disorders [2,3]. However, the cellular and molecular relationships between infections outside the CNS and potential neuronal loss within the CNS is elusive. It is known that in response to certain environment toxins, macrophages/microglia can enter into an overactivated state and release inflammatory cytokines and reactive oxygen species (ROS) that cause neurotoxicity. Lipopolysaccharide (LPS), a major constituent of gram-negative bacteria, is a general activator of immune cells, including microglia and macrophages. LPS induces expression of pro-inflammatory cytokines such as tumor necrosis factor-alpha (TNF-α), interleukin-1β (IL-1β) and IL-6 by microglia [4,5]. These pro-inflammatory cytokines have direct or indirect neurotoxic properties, contributing to neuronal injury [6]. LPS also can induce ROS production in macrophages [7-9]. Microglial activation by LPS plays an important role in the progressive and selective loss of dopaminergic (DA) neurons [10,11]. Microglia-derived superoxide contributes to about 50% of LPS-induced DA neurotoxicity [12,13].

Although microglia are vital in the inflammatory process in the CNS, they may have less chance to be activated during a peripheral bacterial infection, as LPS may not be able to enter the CNS due to the blood-brain barrier (BBB). On the contrary, macrophages in peripheral systems have a greater chance to contact bacterial endotoxins, including LPS, and thus become activated. LPS-activated macrophages can overexpress pro-inflammatory cytokines that enter the CNS, leading to an inflammatory environment. In addition, activated monocytes have the ability to migrate into the CNS, causing neuronal injury. Further, exposure of macrophages/microglia to invading pathogens could lead to the induction of ROS, which can benefit the clearance of pathogens, but on the other hand, cause irreparable damage to bystander neurons [14].

The Bowman-Birk inhibitor (BBI) is a soybean-derived protease inhibitor that has the ability to inhibit trypsin and chymotrypsin activities [15]. BBI is present in many commercial soy foods, such as soymilk, soy-based infant formula, tofu and bean curd. BBI has been shown to have anti-inflammatory effect in both *in vitro *and *in vivo *[16-18]. BBI has an immunoregulation effect through inhibition of proteases released from inflammation-mediating cells [19]. BBI reduces autoimmune inflammation and attenuates neuronal loss in a mouse model of multiple sclerosis, thus ameliorating clinical experimental autoimmune encephalomyelitis [20]. Because inflammation is an important player in macrophage/microglia-mediated neuronal injury, we sought to determine whether BBI has the ability to inhibit LPS-mediated macrophage activation, thus reducing release of pro-inflammatory cytokines and subsequent neurotoxicity in primary cortical neural cultures.

## Methods

### BBI

Bowman-birk inhibitor (BBI) was purchased from Sigma-Aldrich (Cat # T9777). The product is isolated from Glycine max (soybean) and purified from crude trypsin inhibitor (Sigma Cat # T9128). It consists of up to 90% protein as assayed by Biuret, with the remainder a phosphate buffer salt. The concentration used in this study is 1-100 μg/ml (equal to 113.9 nm-11.3 μM).

### Rat cortical neural cultures

Mixed cortical neural cultures were prepared from fetal Sprague Dawley rat embryos at 17-19 days gestation [21]. Dissociated cortical cells were plated in poly-L-lysine coated 96-well plates at 2 × 10^4 ^cells per well or in 24-well plate at 5 × 10^5 ^cells per well in neurobasal media containing the serum and estrogen-free B27 supplement (Gibco BRL, Gaithersburg, MD). Cultures were maintained at 37°C in a humidified 5% CO_2 _atmosphere for two weeks prior to experimentation with medium changed no more than once a week (50% liquid replacement).

### Monocyte-derived macrophage cultures

Monocytes were obtained from the Center for AIDS Research of the University of Pennsylvania School of Medicine. The Center has IRB approval for blood collection from healthy donors. Monocytes were isolated by elutriation; the purity of isolated monocytes is higher than 95%. Blood samples were screened for common blood-born pathogens and certified to be pathogen-free. Freshly isolated monocytes were resuspended in DMEM supplemented with 10% fetal bovine serum (FBS), penicillin (100 U/ml), streptomycin (100 μg/ml) and 1% non-essential amino acids. Cells were cultured in 48-well plates (Corning CellBIND Surface, Corning Incorporated, Corning, NY) at 2.5 × 10^5 ^cells per well. The medium was half-changed every two days. After culture for 7 days, monocytes differentiated into macrophages. Macrophages were first incubated with or without BBI for 24 h and then further treated with LPS for additional 24 h. Supernatants collected from the cell cultures were used to treat rat cortical neurons. Macrophages were lysed in 0.5 mL Tri-reagent (Molecular Research Center, Cincinnati, OH) for total RNA extraction.

### Assessment of neurotoxicity

Neurotoxicity was examined by a cell-based ELISA method which has been successfully used for measuring macrophage-mediated neurotoxicity [22-25]. Briefly, rat cortical neurons cultured in 96-well plates were treated with supernatant from LPS- (1-100 ng/mL) and/or BBI- (1-100 μg/mL) treated macrophage cultures. To block the neurotoxicity of N-methyl-D-aspartic acid (NMDA; Tocris Bioscience; Ellisville, MO), neural cultures were pretreated with (+)-5-methyl-10,11-dihydro-5*H*-dibenzo-cyclohepten-5,10-imine maleate (MK801; Sigma-Aldrich; St. Louis, MO) for 1 h. After 24 h treatment, cells were washed with PBS containing Ca^2+^/Mg^2+ ^and then fixed in 4% paraformaldehyde/4% sucrose for 1 h at room temperature, followed by 1 h blocking in Block A (1 × MEM, 10% FBS, 1 × penicillin/streptomycin, 15 mM HEPES). Cells were then incubated with mouse monoclonal anti-MAP-2 antibody (Sigma-Aldrich, St. Louis, MO) diluted in block A (1:1000) overnight at 4°C. After a wash with PBS, goat α-mouse β-lactamase TEM-1 (Molecular Probes, Eugene, OR) conjugate (1:500; 2 μg/mL) was added into each well and incubated for 30 min and then with fluorocillin green substrate (Invitrogen, Carlsbad, CA) solution in PBS (1 μg/mL) for 1 h. Fluorescence was read at 485/527 nm in a fluorescence microplate reader (PerkinElmer 1420 Multilabel Counter). The fluorescence of untreated neurons (control) was defined as 100%.

### Immunofluorescence staining

Rat cortical cells were seeded on poly-L-lysine coated cover slips in 24-well plates and cultured for two weeks before treatment with supernatant from LPS-activated macrophage culture. After treatment, cells were washed with PBS three times and fixed in ice-cold methanol for 5 min. Non-specific sites were blocked in Block A for 30 min. Cells were then incubated in mouse anti-MAP-2 antibody (1:100) for 1 h, followed by Alexa 488-conjugated anti-mouse IgG for 30 min. After Hoechst (2 μg/mL) staining, the coverslips were mounted on glass slide and observed under a fluorescence microscope (Olympus IX71).

### Reactive oxygen species (ROS) detection

Macrophages were pretreated with or without BBI for 24 h and then incubated with LPS for an additional 24 h. Cells were then washed with serum-free medium, 2'7'-dichlorofluorescin diacetate (DCFH_2_DA; Molecular Probes), which was then added to the cultures and incubated at 37°C for 30 min. ROS production was assessed using a fluorescence microscope (Olympus IX71) at 488 nm.

### Quantitative real-time RT-PCR

Total RNA was extracted with Tri-reagent and reverse transcription was performed using the AMV transcriptase and RNasin (Promega Co., Madison, WI) according to the manufacturer's instructions. The followings primers derived from the published cDNA sequences were used for the PCR amplifications: TNF-α forward, 5'-ATG AGC ACA GAA AGC ATG ATC-3'; TNF-α reverse, 5'-TAC AGG CTT GTC ACT CGA ATT-3'; IL-1β forward, 5'-AAG CTG ATG GCC CTA AAC AG-3'; IL-1β reverse, 5'-AGG TGC ATC GTG CAC ATA AG-3'; IL-6 forward, 5'-AGG AGA CTT GCC TGG TGA AA-3'; IL-6 reverse, 5'-CAG GGG TGG TTA TTG CAT CT-3'; IL-10 forward, 5'-CTT TAA TAA GCT CCA CGA GAA AGG C-3'; IL-10 reverse, 5'-CAG ATC CGA TTT TGG AGA CC-3'; GAPDH forward, 5'-GGT GGT CTC CTC TGA CTT CAA CA-3'; GAPDH reverse, 5'-GTT GCT GTA GCC AAA TTC GTT GT-3'. The oligonucleotide primers were synthesized by Integrated DNA Technologies, Inc. (Coralville, IA). PCR was performed with Brilliant SYBR Green Master Mix (Bio-Rad Laboratories, Hercules, CA) as described previously [26]. All values were calculated using the delta delta Ct method and expressed as change relative to expression of GAPDH mRNA.

### ELISA

TNF-α and IL-6 gene expressions, identified from real time PCR, were evaluated for protein expression using ELISA. After macrophages were treated as indicated in the figure, conditioned medium was collected and levels of TNF-α and IL-6 were measured using conventional double sandwich ELISA kits from Invitrogen Inc. (Carlsbad, CA). Assays were performed according to the manufacturer's instructions.

### Statistical analysis

Data are expressed as the mean ± SD for at least three independent experiments. Statistical significance was analyzed using Student's t-test to compare the means of two groups. For comparison of means of multiple groups, one-way analysis of variance (ANOVA) was performed followed by post Newman-Keuls test. Differences were considered to be statistically significant when the *p*-value was less than 0.05.

## Results

### BBI treatment reduces neurotoxicity of LPS-activated macrophages

**Figure 1:**
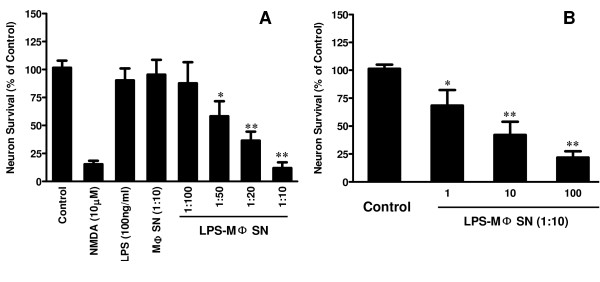
**LPS-activated macrophage (LPS/MΦ) supernatant induces neurotoxicity**. (A) Seven-day-cultured macrophages were treated with or without LPS (100 ng/ml) for 24 h and supernatants collected from the cell cultures were then used to treat rat cortical neurons for 24 h. The percentage of supernatant added to the neuron cultures is indicated. In addition, neuron cultures were treated with either neurobasal media only (control) or media plus NMDA (10 μM) or plus LPS (100 ng/ml). Supernatants collected from untreated and donor-matched macrophage cultures (MΦ SN) were also used as negative controls. (B) Neurotoxicity of activated macrophages treated with different concentrations of LPS. Seven-day-cultured macrophages were treated with different concentrations (1-100 ng/ml) of LPS, and supernatant (1:10) was used to treat rat cortical neuron cultures. The neuron marker MAP-2 was measured by a cell-based ELISA method. Data are expressed as mean ± SD for three independent experiments. (*P < 0.05, **P < 0.01, LPS/MΦ SN vs MΦ SN).

**Figure 2:**
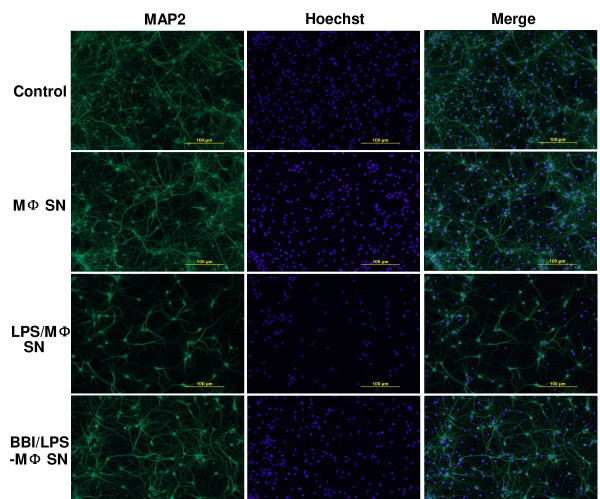
**Immunofluorescence assay of neuronal loss induced by LPS-activated macrophages (MΦ)**. Rat cortical neurons were treated either with complete neurobasal medium (control) or with supernatant from unactivated donor-matched macrophage (MΦ SN), from LPS-activated macrophage supernatant (LPS/MΦ SN), or from BBI-treated and LPS-activated macrophage supernatant (BBI/LPS/MΦ SN) for 24 h. Cells were then washed with PBS and fixed in ice-cold methanol. Cells were incubated with mouse anti-MAP-2 antibody (1:100) for 1 h, then with Alexa 488-conjugated anti-mouse IgG for 30 min. Scale bars: 100 μm.

**Figure 3:**
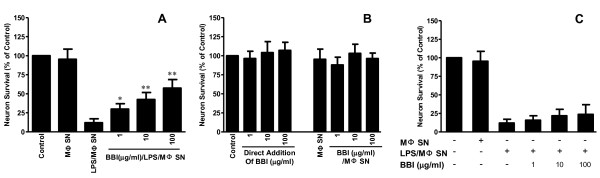
**Impact of BBI on rat cortical neuron cultures**. (A) Effect of BBI on LPS/MΦ-mediated neurotoxicity. Supernatant either from LPS- (100 ng/ml) activated macrophage cultures (LPS/MΦ SN) or from BBI- (1-100 μg/ml) treated and LPS-activated macrophage culture (BBI/LPS/MΦ SN) was used to treat rat cortical neurons (1:10). Untreated and unactivated macrophage supernatant was used as a negative control (MΦ SN). Data are expressed as mean ± SD for three independent experiments. (*P < 0.05, **P < 0.01, BBI/LPS/MΦ SN vs LPS/MΦ SN without BBI). (B) Effect of BBI on neuron death for rat cortical neurons. Rat cortical neurons were directly treated with BBI at the indicated concentrations or with supernatant from either unactivated and untreated macrophage culture (MΦ SN) or BBI- (1-100 μg/ml) treated macrophage culture (BBI/MΦ SN) for 24 h. Data are expressed as mean ± SD for three independent experiments. (C) Effect of BBI treatment on neurotoxicity of LPS-activated macrophages. Cortical neuronal cultures were treated with LPS-activated macrophage supernatant in the presence or absence of BBI for 24 h. Data are expressed as mean ± SD for three independent experiments.

We first examined whether supernatant from LPS-activated macrophage cultures could induce neuron death. Although LPS, when directly added to the rat cortical neuron cultures, had no cytotoxic effect (Figure 1A), supernatant from LPS-activated macrophage cultures induced the neuron death, which was evidenced by decreased MAP-2 expression (Figure 1A and 1B). This LPS/macrophage supernatant-mediated neuronal death was positively related to amount of supernatant added to the rat cortical neuron cultures (Figure 1A). In addition, the concentration of LPS used for macrophage activation was positively associated with degree of neurotoxicity of the LPS/macrophage supernatants (Figure 1B). In contrast, supernatant from BBI-pretreated and then LPS-activated macrophage cultures produced reduced neurotoxicity, compared to that from non-BBI-pretreated cultures (Figure 2 and Figure 3A). Immunofluorescence assays also demonstrated that BBI pre-treatment of macrophages could alleviate the neurotoxicity of LPS-activated macrophages (Figure 2). The direct addition of supernatant from BBI-treated macrophage cultures or of BBI to the neuronal cultures had no cytotoxic effect (Figure 3B). In addition, BBI treatment of neuronal cells had no protective effect against the neurotoxicity of supernatant from LPS-activated macrophage cultures (Figure 3C).

### BBI inhibits LPS-induced inflammatory cytokines

**Figure 4:**
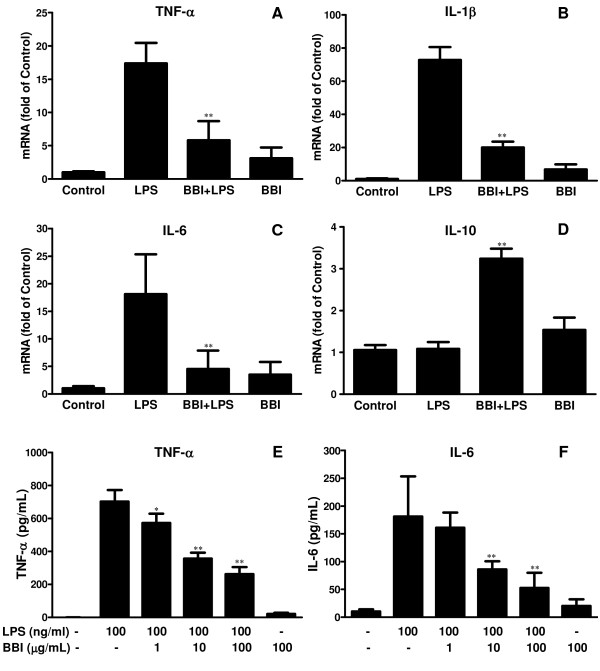
**Effect of BBI on LPS-mediated cytokine expression**. Macrophages were preincubated with or without BBI (100 μg/mL) for 24 h and then treated with LPS (100 ng/mL) for an additional 24 h. (A-D) Total RNA extracted from the cells was subjected to real time RT-PCR mRNAs for cytokine (TNF-α, IL-6, IL-10 and IL-1β) mRNA expression. Data are expressed as mean ± SD for three independent experiments. (*P < 0.05, **P < 0.01, LPS + BBI treatment vs LPS treatment only). (E,F) Supernatants from macrophage cultures with indicated treatments were collected for ELISA measurement of protein levels of TNF-α and IL-6. Data are expressed as mean ± SD for three independent experiments. (*P < 0.05, **P < 0.01, LPS + BBI treatment vs LPS treatment only).

To examine the mechanisms involved in BBI-mediated inhibition of LPS-activated macrophages, we examined whether BBI has the ability to inhibit the expression of inflammatory cytokines induced by LPS. As shown in Figure 4, LPS-treatment of macrophages resulted in induction of TNF-α, IL-1β and IL-6 (Figures 4A,B and 4C). This LPS-mediated induction of cytokines, however, was attenuated by pre-treatment of macrophages with BBI (Figure 4). We also examined cytokine production in the supernatants used to treat cortical neurons. Figure 4E and 4F show that LPS-induced release of TNF-α and IL-6 into the culture supernatant was significantly inhibited by BBI treatment. Although BBI or LPS treatment alone had little effect on IL-10 expression in macrophages, BBI pretreatment induced IL-10 expression in LPS-activated macrophages (Figure 4D).

### BBI inhibits LPS-induced oxidative stress

**Figure 5:**
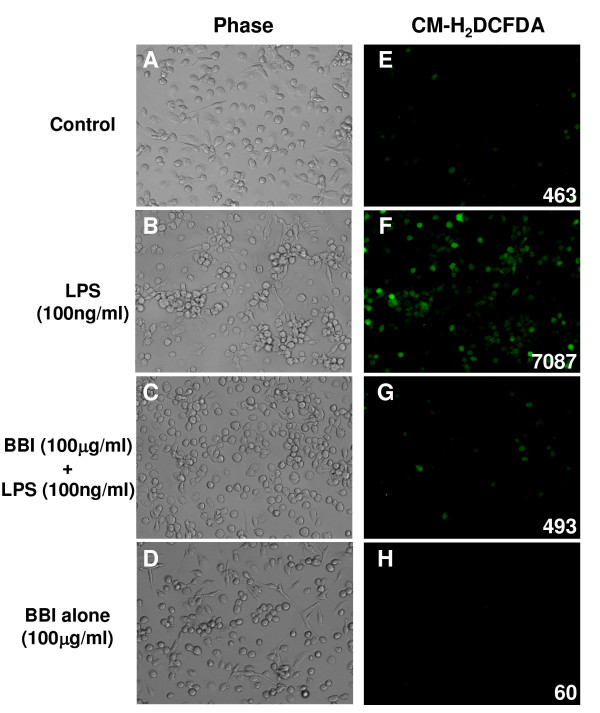
**Effect of BBI on LPS-induced cell aggregation and production of reactive oxygen species (ROS)**. Macrophages were preincubated with or without BBI (100 μg/mL) for 24 h and then treated with LPS (100 ng/mL) for an additional 24 h. Cells were washed with serum-free medium and DCFH_2_DA was then added to the cultures, which were further incubated at 37°C for 30 min. ROS production in macrophages was examined using a fluorescence microscope (magnification: х100). Macrophage aggregation was assessed using a phase contrast microscope. Images presented are representative of three independent experiments. Values on the images indicate fluorescence intensity, as quantified by Image J 1.43.

Activated macrophages/microglia can produce ROS that cause neurotoxicity [3]. Thus, we examined whether BBI treatment of macrophages could reduce ROS production in LPS-activated macrophages. As shown in Figure 5, BBI pretreatment of macrophages significantly attenuated ROS production in LPS-activated macrophages. Morphologically, LPS, when added to macrophage cultures, induced cell aggregation (Figure 5B). However, BBI pretreatment of macrophages suppressed LPS-induced cell aggregation (Figure 5C). BBI treatment alone had no effect on macrophage aggregation (Figure 5D). The aggregated macrophages were highly positive for DCFH_2_DA (indicative of ROS production) (Figure 5B and 5F). BBI treatment significantly reduced LPS-induced ROS production of macrophages as indicated by the fluorescence intensity quantitated by Image J (Figure 5E-H).

### BBI has little effect on NMDA-mediated neuronal death

**Figure 6:**
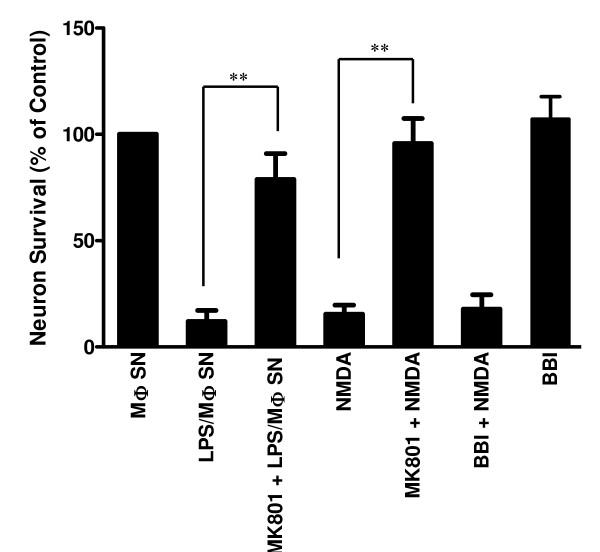
**Effect of BBI on NMDA-induced neuronal death**. Rat cortical neurons were incubated with or without MK801 (5 μM) and/or BBI (100 μg/ml) for 1 h and then treated with LPS- (100 ng/ml) activated macrophage supernatant (LPS/MΦ SN) or NMDA (10 μM) for 24 h. Unactivated donor-matched macrophage supernatant- (MΦ SN) treated cortical neuron cultures were used as controls. Data are expressed as mean ± SD of three independent experiments. (**P < 0.01)

We also determined whether BBI has the ability to reduce NMDA-mediated neuronal death. NMDA, when added to rat cortical neuron cultures, resulted in neuron death (Figure 6). MK801, a NMDA receptor antagonist, could completely block this NMDA-induced cell death (Figure 6). Pretreatment of rat cortical neurons with MK801 could also block LPS/macrophage supernatant-induced neurotoxicity (Figure 6). However, pretreatment of rat cortical neurons with BBI had no effect on NMDA-induced neurotoxicity (Figure 6).

## Discussion

In the present study, we demonstrate that BBI inhibits the neurotoxicity of LPS-activated macrophages. Because activated macrophages can produce inflammatory cytokines that cause neuronal injury, we examined whether BBI treatment of LPS-activated macrophages could inhibit the expression of TNF-α, IL-6 and IL-1β, which are known to be toxic to neurons [22,27,28]. The suppression of these cytokines' production by BBI provides a sound mechanism for BBI-mediated neuroprotection. In addition, BBI/LPS-treated macrophages expressed increased levels of IL-10, a known anti-inflammatory cytokine [29]. It is well known that LPS can activate the nuclear transcription factor NF-κB, leading to the induction of several proinflammatory cytokines in macrophages [30,31]. BBI has the ability to inhibit LPS-induced iNOS/NO and COX-2, which are triggers of NF-κB activation [16]. Therefore, the inhibition of proinflammatory cytokine expression by BBI could be due to its ability to inhibit NF-κB activation. It is also likely that BBI's effect on IL-10 may involve a negative regulation of TLR4/LPS signalling, such as reduction of the production of programmed cell death protein 4 (PDCD4). Recently, PDCD4 was found to promote activation of NF-κB, suppressing IL-10 expression [32].

Despite extensive research on neurodegenerative diseases, the mechanisms of neurodegeneration remain to be determined. One accepted mechanism is that microglia activation by environmental factors is responsible for neuronal injury [33-36]. In addition to resident microglia in the CNS, peripheral macrophages infiltrating into the CNS also play a role in neuroinflammation and neuronal loss under several pathological conditions [37]. Several studies have demonstrated that microglial activation by stimuli such as LPS, amyloid β (Aβ) or TNF-α is toxic to neurons [38,39]. Plasma LPS levels are dramatically increased in certain pathological conditions including sepsis, inflammatory bowel disease, and HIV infection [40]. LPS triggers monocyte/macrophage activation through CD14 and TLR4-mediated signalling, resulting in release of inflammatory cytokines. During neurodegeneration and neurodevelopment, inflammatory cytokines play an important role in the modulation of neuronal survival [22]. The neurotoxic potential of inflammatory cytokines, such as IL-1β, IL-6 and TNF-α, in the CNS has been extensively documented [41]. Experimentally, LPS has been extensively used as a microglia/macrophage activator for the induction of inflammatory dopaminergic neurodegeneration in animal models of Parkinson's disease [2].

Although the mechanisms involved in the anti-inflammatory actions of BBI remain to be determined, the nature of BBI as a serine protease inhibitor explains its ability to inhibit pro-inflammatory cytokine production, as serine proteases induce release of pro-inflammatory cytokines in epithelial cells [42,43] and macrophages [44]. Neurophil-derived serine proteases could cause non-infectious inflammatory processes [45,46]. The serine protease inhibitor (FK-706, α1-antitrypsin) attenuates chemotactic cytokine production in human lung fibroblasts *in vitro *[47] and in human whole blood *in vivo *[48]. The role of serine protease in the induction of proinflammatory cytokines has been further confirmed by a recent study [49] demonstrating that Aβ-induced neurotoxicity is greatly attenuated in serine racemase knockout mice compared to wild type mice.

In summary, we provide compelling experimental evidence that BBI, through inhibition of proinflammatory cytokine production and induction of IL-10, attenuates LPS/macrophage-induced neurotoxicity. BBI also inhibited ROS production, which reduced macrophage aggregation and activation. Since there is lack of effective treatments for neurological disorders, to explore natural products such as BBI as potential treatments for inflammation-mediated neuronal injury is of great interest. Our data support the need of future studies for the development of BBI-based supplementary therapy for the treatment of neuroinflammation and neurodegeneration.

## Abbreviations

BBI: Bowman-Birk inhibitor; LPS: lipopolysaccharide; NMDA: N-methyl-D-aspartic acid; TNF-α: tumor necrosis factor alpha; IL: interleukin; CNS: central nervous system; BBB: blood-brain barrier; HAD: HIV-associated dementia; MK801: (+)-5-methyl-10,11-dihydro-5*H*-dibenzo-cyclohepten-5,10-imine maleate; MΦ: macrophage; MAP-2: microtubule-associated protein 2; RT-PCR: reverse transcriptase-polymerase chain reaction; GAPDH: Glyceraldehyde 3-phosphate dehydrogenase; ELISA: enzyme-linked immunosorbent assay; ANOVA: one-way analysis of variance; Aβ: amyloid β; HAD: HIV-associated dementia; FBS: fetal bovine serum; PBS: phosphate-buffered saline.

## Competing interests

The authors declare that they have no competing interests.

## Authors' contributions

JL designed and performed experiments, and drafted the manuscript. LY, XW and JL performed experiments. DRC performed experiments and co-conceived of the study. DLK co-conceived of the study. WZH conceived of the study, participated in its design and coordination, and drafted the manuscript. All authors have read and approved the final version of this manuscript.
